# Have You Seen the NEWS Today? - a QI Project

**DOI:** 10.1192/bjo.2022.281

**Published:** 2022-06-20

**Authors:** Harleen Kaur Birgi

**Affiliations:** East London NHS Foundation Trust, London, United Kingdom

## Abstract

**Aims:**

The main focus of this QIP was to improve the documentation of NEWS (National early warning scores) and subsequent escalation as appropriate in an Old Age Psychiatric Ward setting. This would in turn lead to improved Physical health outcomes, especially in the COVID-19 pandemic.

**Methods:**

The NEWS chart is based on a simple aggregate scoring system in which a score is allocated to physiological measurements, when patients present to, or are being monitored in hospital. This will ensure that patients who are deteriorating, or at risk of deteriorating, will have a timely initial assessment. This should supplement clinical judgement in assessing the patient's condition.

Early detection and escalation of deteriorating NEWS leads to improved patient outcomes and referral to the appropriate specialties, for subsequent management.

The initial phase of the QIP comprised of retrospective data collection surrounding the recognition and documentation of NEWS on an 18-bedded Old age Psychiatric ward. This period spanned the 2nd wave of the pandemic, from November- December.

Potential interventions were implemented in the form of raising NEWS awareness by educating nursing staff via teaching sessions, displaying posters all over the ward and nursing station. Team also reviewed all NEWS charts everyday during ward management rounds which served as a daily reminder for the staff measuring the observations.

NEWS of & greater than 3 was defined as the threshold for escalation.

Following change implementation, data were collected to capture the progress made over a month.

**Results:**

Analysis of data pre and post- interventions displayed a significant improvement in escalation of unwell patients from 26% to 60%.

**Conclusion:**

Improved outcomes and early detection of potentially deteriorating patients, leading to early transfer of patients to an Acute Medical setting and better overall management.

Raised awareness and understanding of physical health management in Mental Health nurses.

The QIP was presented at the Trust QI Forum meeting and was met by and overwhelmingly positive response. In order to enhance NEWS recording an electronic format is now being adapted. There is also a consideration around providing regular NEWS teaching sessions to all inpatient staff across the trust.

